# Video Head Impulse Test in the Diagnosis of Benign Paroxysmal Positional Vertigo: A Systematic Review of the Literature

**DOI:** 10.3390/audiolres16050154

**Published:** 2026-10-08

**Authors:** Luigi Califano, Salvatore Martellucci, Maria Grazia Melillo, Giuseppe Santopietro

**Affiliations:** 1 Audiology and Phoniatrics Department, AORN San Pio, Via dell’Angelo 1, 82100 Benevento, Italy; luigi.califano1958@gmail.com (L.C.); grazia601@live.com (M.G.M.); 2 ENT Unit, Santa Maria Goretti Hospital, Azienda USL di Latina, 04100 Latina, Italy; dott.martellucci@gmail.com

**Keywords:** benign paroxysmal positional vertigo (BPPV), positional vertigo, video head impulse test (vHIT), vestibulo-ocular reflex, VOR gain

## Abstract

**Background:** Benign paroxysmal positional vertigo (BPPV) is the most common peripheral vestibular disorder and is typically diagnosed based on characteristic positional nystagmus. Although video head impulse testing (vHIT) is increasingly used in vestibular disorders, its role in BPPV remains unclear, particularly in less common variants. This systematic review and meta-analysis aimed to investigate vestibulo-ocular reflex (VOR) gain measured by vHIT in BPPV and to describe vHIT findings in less common variants. **Methods:** A systematic literature search identified studies reporting vHIT findings in patients with BPPV. For the quantitative synthesis, studies including at least 35 patients with common BPPV variants were eligible, and posterior semicircular canal (PSC) VOR gain was analysed using a random-effects meta-analysis. Less common variants were considered separately without a minimum sample-size criterion and qualitatively synthesised, including case reports, case series, and retrospective and prospective studies. **Results:** Ten studies met the inclusion criteria for the quantitative synthesis, while one study about typical BPPV underwent qualitative analysis and six studies investigated less common BPPV variants; one of these six studies also contributed to the quantitative synthesis. In typical PSC-BPPV, vHIT generally showed preserved vestibulo-ocular reflex function, with VOR gain within or close to the normal range. The pooled analysis showed no clinically relevant reduction in PSC VOR gain. In less common variants, vHIT findings were heterogeneous, although most patients showed preserved canal-specific VOR function. **Conclusions:** vHIT appears to have a limited role in the diagnosis of typical BPPV. However, it may provide additional diagnostic information in less common variants, where vestibular responses can be atypical. Further studies with larger and more homogeneous populations are needed to clarify the diagnostic and pathophysiological significance of vHIT findings in uncommon BPPV variants.

## 1. Introduction

Benign paroxysmal positional vertigo (BPPV) is the most common cause of peripheral vertigo and one of the most prevalent vestibular disorders in the general population [1,2,3]. According to the systematic review by Fancello et al. [3], vertigo represents a frequent complaint among older adults, with BPPV accounting for a substantial proportion of vestibular diagnoses in this population, highlighting its considerable epidemiological and clinical impact.

BPPV results from the displacement of otoconia from the utricular macula into one or more semicircular canals. Two principal pathophysiological mechanisms have been described: canalolithiasis, characterised by free-floating otoconia within the lumen of the affected canal, and cupulolithiasis, in which otoconial particles adhere to the ampullary cupula [4,5,6]. In canalolithiasis, displaced otoconia generate abnormal endolymphatic flow and cupular deflection, whereas in cupulolithiasis the increased mass of the cupula modifies its response to gravitational forces. In both conditions, abnormal mechanical stimulation of the ampullary sensory epithelium induces inappropriate activation of the vestibulo-ocular reflex (VOR), resulting in paroxysmal positional nystagmus and vertigo. The biomechanical model proposed by Squires et al. further demonstrated that gravitational displacement of otoconial particles within the semicircular canal can generate sufficient endolymphatic flow to deflect the cupula and activate the ampullary receptor, providing a quantitative explanation for the mechanism underlying canalolithiasis [7].

The posterior semicircular canal is the most frequently affected site, followed by the lateral semicircular canal and, less commonly, the anterior semicircular canal [8]. The Bárány Society established diagnostic criteria for the different forms of BPPV [9], which currently represents the international reference standard for diagnosis and classification. Nevertheless, these diagnostic criteria do not include the reversed apogeotropic variant of posterior canal BPPV, which is currently considered the most common recognised variant of posterior canal BPPV [10,11,12].

Although most patients present with typical clinical features, BPPV represents a heterogeneous disorder encompassing several clinical and pathophysiological variants. Some of these forms may mimic central vestibular disorders, particularly in patients presenting with persistent positional downbeat nystagmus, in whom distinguishing anterior canal BPPV, apogeotropic posterior canal BPPV, and cerebellar pathology may be challenging [13,14].

The diagnosis of BPPV is primarily based on the identification of characteristic paroxysmal positional nystagmus elicited by manoeuvres that selectively stimulate the affected semicircular canal. These include the Dix–Hallpike and Semont positioning tests for posterior canal BPPV (PC-BPPV) and the Head-Yaw Test (Pagnini–McClure test) for lateral canal BPPV (LC-BPPV) [15,16,17,18]. Although these manoeuvres provide high diagnostic accuracy in most patients, atypical presentations may require additional functional assessment. Castellucci et al. [14] demonstrated that patients with positional downbeat nystagmus due to apogeotropic posterior canal BPPV may exhibit reduced VOR gain on the Video Head Impulse Test (vHIT), suggesting that such abnormalities may result from altered semicircular canal biomechanics secondary to incomplete canalith jam rather than irreversible vestibular receptor damage.

The Video Head Impulse Test (vHIT) has become one of the most widely used methods for the quantitative assessment of semicircular canal function. Introduced by Ulmer and Chays [19] and subsequently validated by MacDougall et al. using a different recording system [20], vHIT measures vestibulo-ocular reflex (VOR) gain during rapid, high-acceleration head impulses. Unlike caloric testing, which primarily assesses lateral semicircular canal function at very low frequencies, vHIT evaluates all six semicircular canals using high-frequency stimuli that more closely reproduce physiological head movements [19,20,21,22].

During the Head Impulse Paradigm (HIMP), the patient maintains visual fixation on a stationary target while the examiner delivers brief, rapid, and unpredictable head impulses in the plane of the semicircular canal under investigation. In healthy individuals, compensatory eye movements stabilise retinal images, resulting in preserved VOR gain. Conversely, reduced VOR gain and the presence of covert and/or overt corrective saccades indicate impaired high-frequency semicircular canal function [20,21,22].

Because BPPV is primarily a mechanical disorder rather than a structural lesion of the vestibular sensory epithelium or vestibular nerve, high-frequency semicircular canal function would be expected to remain preserved in most patients. Consequently, abnormal vHIT findings may suggest disturbed semicircular canal mechanics or concomitant vestibular pathology [23,24,25,26,27,28,29,30,31,32,33,34].

Several studies have reported abnormal vHIT findings in selected BPPV variants, particularly in patients with canalith jam, associated spontaneous nystagmus, or atypical involvement of the vertical and lateral semicircular canals [14,32,35,36,37,38,39]. These observations suggest that vHIT abnormalities may reflect altered semicircular canal biomechanics, resulting in altered endolymphatic hydrodynamics and impaired transmission of mechanical forces to the cupula, rather than permanent vestibular receptor dysfunction. Therefore, vHIT may provide complementary functional information alongside positional testing in patients with atypical clinical presentations or suspected disturbances of semicircular canal mechanics.

Despite the growing number of published studies, the evidence regarding the role of vHIT in BPPV remains fragmented. Reported findings vary according to the clinical subtype, affected semicircular canal, disease stage, underlying pathophysiological mechanism, and methodological characteristics of individual studies. Furthermore, the clinical significance of reduced VOR gain and corrective saccades in BPPV has not yet been comprehensively established. Although vHIT is generally considered of limited diagnostic value in uncomplicated BPPV, reduced VOR gain has been reported in selected patients, particularly in secondary or concomitant forms of BPPV and in the presence of associated vestibular dysfunction. For example, Liu et al. demonstrated a reduction in VOR gain measured by vHIT in patients with BPPV secondary to sudden sensorineural hearing loss [40]. These apparently discrepant findings raise the question of whether reduced VOR gain represents an intrinsic feature of BPPV or is instead related to specific clinical subtypes, altered semicircular canal biomechanics, or concomitant vestibular pathology.

Therefore, the aim of the present systematic review and meta-analysis was to critically evaluate the available evidence regarding vHIT findings in patients with BPPV, including both typical and atypical variants. Specifically, we investigated whether abnormalities of VOR gain are associated with particular clinical and pathophysiological subtypes of the disorder and examined the relationship between vHIT findings and alterations in semicircular canal biomechanics, with particular attention to canalith jam and other atypical positional nystagmus patterns. Finally, we explored the potential clinical role of vHIT as a complementary tool for the functional assessment and differential diagnosis of patients with BPPV.

## 2. Materials and Methods

A systematic review of the literature was conducted to evaluate the vHIT findings, functional assessment, and clinical application of the Video Head Impulse Test (vHIT) in patients with benign paroxysmal positional vertigo (BPPV). This systematic review and meta-analysis was conducted and reported in accordance with the Preferred Reporting Items for Systematic Reviews and Meta-Analyses (PRISMA) 2020 guidelines [41]. The completed PRISMA checklist is provided as Supplementary Material.

The literature search was performed in the PubMed/MEDLINE and Scopus electronic databases and included all studies published up to 31 May 2026, without restrictions regarding the year of publication. Google Scholar was used as a supplementary source to identify potentially relevant studies not retrieved through the primary database search.

For PubMed/MEDLINE, Scopus, and Google Scholar, the same search strategy was used, combining terms related to BPPV/positional vertigo and video head impulse testing. For PubMed/MEDLINE, the following search strategy was used: ((“Benign Paroxysmal Positional Vertigo” [Mesh] OR “Benign Paroxysmal Positional Vertigo” [Title/Abstract] OR BPPV [Title/Abstract] OR “Positional Vertigo” [Title/Abstract]) AND (“Video Head Impulse Test” [Title/Abstract] OR “Video Head Impulse Testing” [Title/Abstract] OR vHIT [Title/Abstract])). Only articles published in English were considered eligible. Titles and abstracts were screened jointly by two reviewers (G.S. and L.C.). Full-text articles were subsequently assessed by the same reviewers, and eligibility was determined by consensus.

The protocol of this systematic review was not prospectively registered in PROSPERO or in any other publicly accessible database. No separate review protocol was prepared.

Studies investigating less common BPPV variants were considered separately and synthesised qualitatively because of their rarity and the heterogeneity of the available evidence The processes of study identification, screening, eligibility assessment, and inclusion of are summarised in the PRISMA flow diagram (Figure 1).

Studies about common BPPV type were included if they fulfilled all of the following eligibility criteria:

- A definite diagnosis of BPPV established according to the diagnostic criteria of the Bárány Society;
- Assessment of vestibular function using the Video Head Impulse Test;
- Reporting of VOR gain values or sufficient quantitative data to allow their calculation;
- A study population including at least 35 patients.

The minimum sample-size threshold of 35 participants was a pragmatic, predefined methodological criterion adopted by the authors to limit the inclusion of small case series and to obtain a more robust quantitative synthesis of vHIT-derived VOR gain values. This threshold was not derived from a formal statistical calculation and was not intended to represent a universally validated minimum sample size for studies of BPPV or vHIT, but rather to improve the comparability and statistical robustness of the studies included in the quantitative analysis.

The following studies were excluded:

- Case reports;
- Narrative or systematic reviews;
- Editorials;
- Letters to the Editor;
- Studies not reporting quantitative VOR gain data;
- Studies including patients with concomitant audiovestibular disorders or vestibular conditions potentially affecting vHIT findings, including Menière’s disease, acute unilateral vestibular loss, sudden sensorineural hearing loss, Lindsay–Hemenway syndrome, vestibular neuritis, or other peripheral or central vestibular disorders.

The prospective study by Manzari and Tramontano [34] was not included in the quantitative synthesis because, although it reported a mean VOR gain for the affected semicircular canals, the VOR gain was not provided separately for patients with PSC and LSC BPPV and therefore could not be specifically attributed to the PSC subgroup included in our meta-analysis. Nevertheless, because the study specifically evaluated patients during the acute phase of BPPV, its findings were retained for the qualitative synthesis. Because several BPPV variants are exceptionally uncommon—including anterior semicircular canal BPPV, apogeotropic posterior canal BPPV, direction-fixed lateral canal BPPV, and canalith jam—the minimum sample-size criterion was not applied to these subgroups, and case reports were accepted too. Studies investigating these rare variants were identified and considered for qualitative assessment; however, owing to the limited number of cases and the substantial clinical heterogeneity, they were analysed descriptively only and were not included in the quantitative meta-analysis.

For each eligible study, the following data were extracted: first author, year of publication, country, study design, sample size, sex distribution, mean age, involved semicircular canal, distinction between canalolithiasis and cupulolithiasis (when reported), mean vestibulo-ocular reflex (VOR) gain values for each semicircular canal, presence of corrective saccades, and caloric test findings. Data extraction was performed jointly by two reviewers (G.S. and L.C.). The extracted data were subsequently checked together by both reviewers to ensure accuracy and consistency.

When studies reported separate subgroups (e.g., geotropic and apogeotropic lateral canal BPPV), subgroup means and standard deviations were combined to obtain a single estimate for the corresponding semicircular canal. Combined means were calculated as weighted averages according to subgroup sample size, whereas pooled standard deviations were derived according to the methods recommended in the Cochrane Handbook for Systematic Reviews of Interventions [42]. When standard deviations were not directly reported but sample size together with minimum and maximum values or interquartile ranges were available, standard deviations were estimated using the method proposed by Wan et al. [43].

The methodological quality of the observational studies included in the quantitative synthesis was assessed using the Newcastle–Ottawa Scale (NOS) [44]. The risk-of-bias assessment was performed for descriptive purposes only and was not used as an exclusion criterion. The risk-of-bias assessment was performed jointly by two reviewers (G.S. and L.C.), with the final assessment determined by consensus. Given the observational nature of the included studies and the substantial methodological heterogeneity, no formal assessment of the certainty of evidence was performed.

Given the heterogeneous study designs and the inclusion of case reports among studies investigating less common BPPV variants, no formal risk-of-bias assessment was performed for these studies; their findings were instead synthesised descriptively.

Separate random-effects meta-analyses of mean VOR gain were performed for the posterior semicircular canal (PSC) and the lateral semicircular canal (LSC). A random-effects model was selected because of the expected clinical and methodological heterogeneity among studies, including differences in BPPV subtypes, affected semicircular canals, patient characteristics, and vHIT protocols.

Because only two studies reported geotropic and apogeotropic lateral canal BPPV separately, these variants were pooled and analysed using a single random-effects model for lateral semicircular canal involvement.

The pooled effect estimate was the mean VOR gain, expressed with corresponding 95% confidence intervals (95% CI). For the pooled VOR gain estimates, *p*-values (where reported) refer to the test of the null hypothesis that the mean gain equals zero and are not clinically informative; interpretation therefore relies on the point estimate, its 95% confidence interval, and the accompanying heterogeneity statistics. Between-study heterogeneity was assessed using Cochran’s Q test and the I^2^ statistic and interpreted according to conventional thresholds (25%, low; 50%, moderate; and 75%, high heterogeneity). No formal subgroup or meta-regression analyses were performed because of the limited number of included studies. However, since only two eligible PSC-BPPV studies had been excluded solely on the basis of the 35-patient threshold, a post hoc sensitivity analysis was performed by adding these two studies to the primary PSC dataset, in order to test the robustness of the pooled estimate to this pre-specified inclusion criterion.

Potential publication bias and small-study effects were assessed by visual inspection of funnel plots, Egger’s regression test, and Kendall’s Tau rank correlation test. These analyses were interpreted cautiously because of the limited number of studies available for some comparisons.

All statistical analyses were performed using Jamovi (version 2.6.45.0; The Jamovi Project, Sydney, Australia) with the MAJOR (Meta-Analysis for Jamovi) module. A two-sided *p*-value < 0.05 was considered statistically significant.

Given that the available evidence regarding vHIT findings in patients with BPPV consists predominantly of retrospective or prospective observational studies, the results of this meta-analysis should be interpreted cautiously, considering the methodological heterogeneity and the observational nature of the included studies.

## 3. Results

### 3.1. vHIT Findings in Common Forms of BPPV

The literature search identified 145 records evaluating the use of the Video Head Impulse Test (vHIT) in patients with benign paroxysmal positional vertigo (BPPV). After removal of duplicate records and screening of titles and abstracts, potentially eligible articles underwent full-text assessment. According to the predefined eligibility criteria, ten studies were included in both the qualitative synthesis and the quantitative meta-analysis (Figure 1).

The included studies were published between 2020 and 2026 and comprised six retrospective studies, three prospective observational studies, and one cross-sectional study. The studies originated from China, India, Italy, Japan, South Korea, Thailand, and Türkiye.

Overall, the included studies evaluated 805 patients with BPPV without associated audiovestibular disorders potentially affecting vestibular function. The pooled population consisted of 320 males (39.8%) and 485 females (60.2%), with ages ranging from 16 to 91 years. The principal demographic, clinical, and methodological characteristics of the included studies are summarised in Table 1.

Regarding the timing of vHIT assessment, all included studies except Kabaya et al. [27] performed vHIT before repositioning manoeuvres and immediately after the diagnostic positional manoeuvres. Califano et al. [24], Vongviriyangkoon et al. [29], and Uslu et al. [31] also performed vHIT after canalith repositioning; however, for the present meta-analysis, the VOR gain values obtained while BPPV was still active, before repositioning, were considered. In these studies, no statistically significant difference was observed between pre- and post-repositioning VOR gain values. Wenyan et al. [30] divided the study population into two groups, with vHIT performed either before or after repositioning manoeuvres; as no statistically significant difference was found between the two groups, all patients were included in the present meta-analysis.

The study by Song et al. [32] included patients with lateral semicircular canal (LSC) cupulolithiasis and light cupula syndrome. Only patients with LSC cupulolithiasis were included in the quantitative analysis of typical BPPV, whereas patients with light cupula syndrome were analysed separately within the descriptive review of uncommon BPPV variants.

Posterior semicircular canal BPPV (PSC-BPPV) was the most frequently investigated subtype and was represented in 8 of the 10 studies included in the quantitative synthesis. Most studies reported posterior canal vestibulo-ocular reflex (VOR) gain values within the normal range. Mild reductions in VOR gain of the affected posterior canal were described in some cohorts, whereas others found no significant differences between the affected and contralateral sides.

Corrective saccades were reported in several studies and were observed both in patients with preserved VOR gain and in those showing mild reductions of gain. When reported, covert and overt saccades were generally confined to the affected posterior semicircular canal.

Among the ten studies included in the quantitative synthesis, only two—Califano et al. [24] and Kim et al. [25]—restricted enrolment to the acute phase of BPPV (symptom onset ≤ 1 week, first episode). In these genuinely acute-phase cohorts, PSC VOR gain was 0.75 ± 0.15 and 0.92 ± 0.17 respectively, both within or close to the range considered compatible with preserved high-frequency function. Califano et al. [24] also identified a subgroup (6.7% of patients) with transiently reduced gain that normalised after resolution of the acute phase. Importantly, the qualitative evidence also included the prospective study by Manzari and Tramontano [34], which specifically assessed 90 patients with BPPV during the acute phase. Eighty patients had posterior canal involvement while 10 patients had LSC-BPPV. vHIT showed no reduction in VOR gain in the examined semicircular canals (0.91±0.07), supporting preserved high-frequency canal function during the acute phase. Because the study reported VOR gain for the affected semicircular canals overall rather than separately for the PSC subgroup, its findings were not included in the quantitative PSC meta-analysis but were considered in the qualitative synthesis. The remaining studies enrolled patients with highly variable disease duration—from a few days to several months or, in some cohorts, years [27,28]—without stratifying VOR gain by time since onset.

Evidence regarding lateral semicircular canal BPPV (LSC-BPPV) was more limited than for PSC-BPPV. Because only two studies reported separate results for geotropic and apogeotropic variants, both subtypes were pooled for quantitative analysis.

Most studies reported lateral canal VOR gain values within the normal range. Slight reductions in VOR gain on the affected side were described in some patient cohorts, whereas corrective saccades were reported in a proportion of patients, irrespective of the geotropic or apogeotropic presentation.

Only a limited number of studies systematically reported caloric test findings. Available data showed variable caloric responses, without a consistent relationship between caloric abnormalities and vHIT findings. In the studies reporting both examinations, reduced caloric responses were not consistently associated with decreased VOR gain on vHIT.

The random-effects meta-analysis of PSC-BPPV demonstrated a pooled mean posterior canal VOR gain of 0.81 (95% CI: 0.73–0.89) (Figure 2). Between-study heterogeneity was high (Cochran’s Q = 241.710, *p* < 0.001; I^2^ = 97.03%).

A sensitivity analysis including the two PSC-BPPV studies excluded solely due to the sample-size threshold (Fallahnezhad et al. [45], *n* = 29; Çınar et al. [46], *n* = 24) yielded a pooled VOR gain of 0.81 (95% CI: 0.73–0.88; I^2^ = 97.1%) virtually unchanged from the primary estimate (0.81; 95% CI: 0.73–0.89; I^2^ = 97.03%), indicating that the pre-specified threshold did not materially influence the pooled result. Fallahnezhad et al. [45], in a pilot study of 29 patients with unilateral PSC-BPPV, reported reduced ipsilesional posterior canal VOR gain (<0.7) in 16 patients (55.2%), with a significant difference between ipsilesional and contralesional gain (0.66 vs. 0.77, *p* = 0.024); no corrective saccades were observed. Çınar et al. [46] evaluated 24 patients with isolated PSC-BPPV before and immediately after CRM and at day 3, day 7 and month 1, and found no significant changes in VOR gain or gain asymmetry over time and no difference from 20 matched controls (mean pre-treatment gain 0.92 ± 0.18; abnormal gain, defined by a control-derived cut-off of 0.68, in 2 patients); no corrective saccades were observed.

The random-effects meta-analysis of LSC-BPPV demonstrated a pooled mean lateral canal VOR gain of 0.98 (95% CI: 0.9–1.07) (Figure 3). Geotropic and apogeotropic variants were analysed together because only two studies provided separate subgroup data. Between-study heterogeneity was high (Cochran’s Q = 102.848, *p* < 0.001; I^2^ = 98.55%).

Visual inspection of the funnel plots did not demonstrate marked asymmetry. Similarly, neither Egger’s regression test nor Kendall’s Tau rank correlation test showed statistically significant evidence of publication bias or small-study effects.

All data are summarised in Table 2.

### 3.2. vHIT Findings in Less Common Forms of BPPV

The predefined sample-size criterion precluded quantitative synthesis of several less common forms of BPPV. These conditions were therefore analysed qualitatively.

Because of the exceptional rarity of several BPPV variants and the predefined minimum sample-size criterion adopted for the quantitative synthesis, studies investigating these conditions were analysed separately within the qualitative review. Owing to the limited number of available patients and the marked clinical and methodological heterogeneity among studies, a quantitative meta-analysis was not considered appropriate.

Six studies investigating uncommon BPPV variants were identified. These articles, published between 2014 and 2026, comprised three retrospective observational studies and three case reports from China, Spain, and Italy.

The overall population included 122 patients, comprising 33 males (27.1%) and 89 females (72.9%), with ages ranging from 18 to 84 years. The principal characteristics of these studies are summarised in Table 3. In all the studies vHIT was performed at the same visit as the diagnostic positional examination, prior to any repositioning manoeuvres.

The analysed conditions included 48 patients (39.3%) with apogeotropic posterior semicircular canal BPPV, 51 (41.8%) with lateral semicircular canal light cupula, 21 (17.2%) with anterior semicircular canal BPPV, and one patient (0.8%) with lateral semicircular canal canalith jam. One additional patient presented positional downbeat nystagmus and could not be definitively classified as either anterior canal BPPV or apogeotropic posterior canal BPPV.

Two studies evaluated vHIT findings in anterior semicircular canal BPPV. Reported anterior canal VOR gain values ranged from normal values (0.85 ± 0.11) to reduced values (0.46–0.62). Corrective saccades were inconsistently reported and, when present, involved the affected anterior semicircular canal.

Studies investigating apogeotropic posterior semicircular canal BPPV reported mean posterior canal VOR gain values ranging from 0.57 ± 0.10 to 0.62 ± 0.11. In selected patients, spontaneous nystagmus and corrective saccades were observed in association with reduced VOR gain.

The largest cohort among the uncommon variants consisted of patients with lateral semicircular canal light cupula. Song et al. [32] reported a mean lateral canal VOR gain of 1.02 ± 0.05, indicating preserved high-frequency semicircular canal function despite the presence of persistent positional nystagmus. Spontaneous nystagmus was documented in 27 of the 51 patients included in the study.

Only one patient with lateral semicircular canal canalith jam fulfilled the eligibility criteria for the qualitative review. This patient showed a reduced lateral canal VOR gain (0.59), associated with spontaneous nystagmus and corrective saccades.

Overall, vHIT findings in uncommon BPPV variants were considerably more heterogeneous than those observed in typical posterior and lateral canal BPPV. While several uncommon variants showed preserved VOR gain, selective reductions of the affected semicircular canal and associated corrective saccades were reported in others, particularly in patients presenting with positional downbeat nystagmus or canalith jam.

Detailed data extracted from the studies about the less common BPPV are summarised in Table 4.

## 4. Discussion

To our knowledge, this is the first systematic review and meta-analysis to provide a quantitative study of vHIT findings in patients with BPPV. The pooled analyses showed mean VOR gain estimates within the range generally considered compatible with preserved high-frequency semicircular canal function in both PSC-BPPV and LSC-BPPV. However, between-study heterogeneity was extremely high (I^2^ = 97% and 99%, respectively), indicating substantial variability across the included studies. Therefore, the pooled estimates should be interpreted as approximate summaries rather than as precise or universally generalisable measures of VOR function in BPPV.

These findings are consistent with the current pathophysiological model of BPPV, which identifies the disorder as a mechanical alteration of semicircular canal hydrodynamics rather than a disease characterised by structural damage to the vestibular sensory epithelium or vestibular nerve. Consequently, the mere presence of displaced otoconial material within the semicircular canal would not be expected, in itself, to impair the high-frequency vestibular responses evaluated by vHIT.

This concept can be explained by considering the different physiological mechanisms explored by positional testing and vHIT. Positional manoeuvres, such as the Dix–Hallpike and Pagnini–McClure tests, provoke gravitational displacement of otoconial particles within the affected semicircular canal, generating abnormal endolymphatic flow and cupular deflection responsible for positional nystagmus. Conversely, vHIT evaluates the dynamic performance of the high-frequency vestibulo-ocular VOR during rapid angular head accelerations. Rather than providing competing information, these examinations therefore investigate complementary aspects of semicircular canal physiology: positional testing demonstrates the mechanical consequences of gravity-dependent otoconial displacement, whereas vHIT evaluates the functional integrity of the vestibular receptor and its neural pathways during high-frequency stimulation.

The biomechanical model proposed by Squires et al. [7] provides a convincing explanation for the apparent discrepancy between these findings. The quantity of otoconial material required to generate pathological endolymphatic flow during slow gravitational movements is likely insufficient to interfere substantially with the transmission of the rapid hydrodynamic forces generated during head impulses. Consequently, abnormal positional nystagmus may coexist with preserved VOR gain. From this perspective, normal vHIT findings should not be interpreted as evidence against the diagnosis of BPPV but rather as a physiological finding compatible with an essentially mechanical disorder. Positional testing and vHIT interrogate fundamentally different stimulus conditions. Positional manoeuvres assess low-frequency, gravity-dependent canal biomechanics, whereas vHIT probes high-frequency angular VOR responses. A canal can therefore show a preserved high-frequency VOR gain on vHIT while simultaneously exhibiting altered gravity-dependent biomechanics sufficient to generate positional nystagmus. Furthermore, in a subset of patients, canalith jam or associated vestibular pathology may transiently affect even the high-frequency response, so that normal or abnormal vHIT findings should be interpreted as reflecting the specific biomechanical or pathophysiological state of the canal at the time of testing, rather than as a general statement about the anatomical or functional integrity of the vestibular receptor as a whole.

The complementary nature of vestibular investigations performed at different stimulus frequencies further supports this interpretation. As demonstrated by Mahringer and Rambold [33], caloric irrigation and vHIT assess distinct frequency domains of semicircular canal function and should therefore be regarded as complementary rather than interchangeable examinations. Similarly, Curthoys et al. [47] emphasised that interpretation of VOR gain requires consideration of semicircular canal anatomy and the geometric principles underlying the head impulse test, reinforcing the concept that preserved vHIT responses do not exclude clinically relevant disturbances of canal biomechanics.

Accordingly, the present review indicates that reduced VOR gain is not an intrinsic characteristic of uncomplicated BPPV. Across studies investigating typical PSC-BPPV and LSC-BPPV, VOR gain values remained consistent with preserved high-frequency semicircular canal function, indicating that freely mobile otoconial debris does not usually compromise the ability of the vestibular system to encode high-frequency angular accelerations. Whenever abnormalities were identified, they were generally confined to specific clinical conditions in which normal semicircular canal biomechanics appeared to be altered. The two PSC-BPPV studies excluded from the quantitative synthesis on sample-size grounds reached discordant conclusions [45,46]. This discrepancy may reflect differences in the gain threshold applied (a fixed cut-off of 0.7 versus a control-derived cut-off of 0.68), in the recording systems and sampling rates, and in study design, since only Çınar et al. included a control group. The interval from symptom onset was not reported by Fallahnezhad et al. and averaged approximately 10 days in Çınar et al. Moreover, Fallahnezhad et al. acknowledged that vertical canal recordings may be affected by artefacts such as eyelid interference. No corrective saccades were observed in either study, irrespective of VOR gain. Taken together with the pooled estimate, these findings are compatible with the view that reduced PSC VOR gain is not a consistent feature of uncomplicated BPPV, although the available data do not allow us to establish whether, when present, it reflects transient mechanical factors or measurement variability.

The available evidence suggests that abnormal vHIT findings may be associated with disorders characterised by disturbed canal biomechanics rather than permanent vestibular receptor dysfunction. Reduced VOR gain may reflect transient mechanical changes capable of modifying the transmission of endolymphatic forces to the cupula during high-frequency stimulation. However, this interpretation should be considered a pathophysiological hypothesis rather than a definitive causal relationship established by the present evidence.

Among these conditions, canalith jam represents the most convincing pathophysiological model. Unlike canalolithiasis, in which otoconial particles remain freely mobile and generate abnormal endolymphatic flow primarily during gravitational head movements, canalith jam is characterised by partial or complete obstruction of the semicircular canal by aggregated otoconial material. Such obstruction may interfere not only with gravity-dependent endolymphatic displacement but also with the transmission of rapid hydrodynamic forces generated during head impulses, thereby potentially producing a reversible reduction in VOR gain.

This hypothesis is supported by several clinical observations. Castellucci et al. [14] first reported selective reduction of VOR gain in the involved semicircular canal in patients presenting with positional downbeat nystagmus, proposing incomplete canalith jam as the most likely mechanism. Subsequently, the same group described spontaneous upbeat nystagmus associated with selective anterior semicircular canal hypofunction, suggesting anterior canal canalith jam as the underlying pathophysiological substrate [35]. Similar observations have been reported in patients presenting with isolated lateral semicircular canal hypofunction mimicking acute unilateral peripheral vestibulopathy [36], as well as in spontaneous lateral canal jamming associated with contralateral posterior canal canalolithiasis [39]. In these clinical situations, the functional deficit detected by vHIT appeared to resolve after elimination of the mechanical obstruction, supporting the concept of transient biomechanical impairment rather than irreversible vestibular injury.

The present review therefore supports a distinction between uncomplicated canalolithiasis and those clinical conditions in which the hydrodynamics of the affected semicircular canal are modified by the configuration or behaviour of the displaced otoconial material. In typical BPPV, otoconia remain sufficiently mobile to generate positional symptoms without significantly affecting the transmission of high-frequency stimuli. Conversely, when endolymphatic flow is mechanically hindered or redirected, reversible impairment of VOR gain may occur despite preservation of the vestibular sensory epithelium.

This interpretation is also consistent with findings reported in several uncommon forms of BPPV included in the qualitative analysis. Although the available evidence remains limited, selective reductions in VOR gain were reported predominantly in patients presenting with persistent positional nystagmus, spontaneous nystagmus, or clinical findings suggestive of disturbed canal hydrodynamics. By contrast, semicircular canal function remained preserved whenever the underlying mechanism was compatible with uncomplicated canalolithiasis.

Taken together, these observations suggest that vHIT primarily reflects the functional consequences of altered semicircular canal biomechanics rather than the mere presence of displaced otoconial debris. Reduced VOR gain should therefore not be regarded as a diagnostic feature of BPPV itself but rather as a potential indicator of mechanical conditions capable of modifying endolymphatic dynamics or of concomitant vestibular disease.

These findings also have important clinical implications. The preservation of high-frequency semicircular canal responses observed in typical PSC-BPPV and LSC-BPPV indicates that vHIT should not be considered a primary diagnostic test for uncomplicated BPPV. Diagnosis remains based on positional testing and identification of characteristic positional nystagmus according to the Bárány Society criteria, whereas vHIT provides complementary information regarding semicircular canal function.

Conversely, abnormal vHIT findings require careful interpretation. Reduced VOR gain is uncommon in uncomplicated BPPV and should prompt consideration of altered canal biomechanics, such as canalith jam, or concomitant vestibular disorders including vestibular neuritis, Menière’s disease, or secondary BPPV associated with inner-ear pathology. Consequently, abnormal vHIT findings should not be attributed automatically to canalolithiasis but should instead prompt a broader vestibular assessment.

Rather than indicating a consistent physiological signal, the marked heterogeneity observed (I^2^ = 97–99%) suggests that pooled VOR gain estimates should be interpreted with caution. Part of this variability may stem from methodological differences—including acquisition protocols, gain calculation algorithms, timing of assessment relative to symptom onset, demographic characteristics, and examiner experience—but a genuine contribution of clinical and pathophysiological diversity across studies cannot be excluded. The overall qualitative pattern (VOR gain generally within or close to the normal range in most individual studies) may therefore be more informative than the single pooled point estimate.

A further potential source of heterogeneity relates to the clinical timing of vHIT assessment relative to symptom onset. Findings obtained during the acute phase of BPPV should not necessarily be considered physiologically equivalent to measurements obtained weeks or months after onset. This interpretation is also supported by the prospective study of Manzari and Tramontano [34], which evaluated patients during the acute phase of BPPV and found preserved VOR gain in the affected semicircular canals, although the study did not provide canal-specific values that could be separately attributed to the PSC subgroup. These findings are consistent with the preserved high-frequency canal function observed in the acute-phase cohorts included in the present quantitative synthesis.

Future research should focus on those clinical conditions in which vestibular function may be transiently or permanently affected, including secondary BPPV associated with vestibular neuritis, Menière’s disease, and other inner-ear disorders. It should be emphasised that the quantitative synthesis specifically addresses uncomplicated, idiopathic BPPV, since studies involving patients with Menière’s disease, vestibular neuritis, sudden sensorineural hearing loss, or other confounding audiovestibular disorders were excluded a priori. Consequently, the pooled findings cannot be extrapolated to secondary or concomitant forms of BPPV, in which the underlying vestibular pathology itself—rather than the otoconial displacement—may account for altered VOR gain. Prospective multicentre longitudinal studies using standardised vHIT acquisition protocols and serial assessments before and after canalith repositioning procedures will be essential to clarify the temporal evolution and reversibility of VOR abnormalities and to further define the contribution of disturbed canal biomechanics, particularly canalith jam, to the pathophysiology of atypical BPPV.

This study has several limitations. First, the number of available studies investigating vHIT in BPPV remains limited, particularly for uncommon variants such as anterior canal BPPV, apogeotropic posterior canal BPPV, and canalith jam, precluding robust quantitative analyses in these subgroups. Second, methodological heterogeneity among the included studies—including differences in recording systems, acquisition protocols, gain calculation algorithms, diagnostic criteria, and timing of assessment—may have influenced the pooled estimates, particularly in light of the marked between-study heterogeneity noted above. Third, most studies were observational and cross-sectional, limiting conclusions regarding the temporal evolution and reversibility of VOR abnormalities following successful canalith repositioning procedures. Fourth, restricting the quantitative synthesis to studies reporting VOR gain values may have introduced selection bias. Finally, the technical and methodological characteristics of vHIT acquisition and analysis may influence VOR gain estimates; therefore, the pooled values should be interpreted primarily as an approximate summary of generally preserved high-frequency vestibular function across individual studies, rather than as establishing a precise, universally applicable numerical VOR-gain value for BPPV. A post hoc sensitivity analysis confirmed that the pre-specified 35-patient threshold did not materially affect the pooled PSC estimate, supporting the robustness of this methodological choice; this could only be tested for the two PSC-BPPV studies identified as excluded solely for this reason during screening.

## 5. Conclusions

This systematic review and meta-analysis demonstrate that BPPV is generally associated with preserved high-frequency semicircular canal function when assessed by vHIT in patients with otherwise uncomplicated, idiopathic BPPV. The pooled VOR gain values observed in PC-BPPV and LC-BPPV are consistent with preserved peripheral vestibular function, supporting the concept that typical BPPV is fundamentally a mechanical disorder of endolymphatic–otoconial dynamics rather than a disease characterised by structural dysfunction of the vestibular sensory apparatus. These pooled estimates should nonetheless be interpreted as an approximate summary of the overall pattern observed across individual studies, rather than as precise quantitative parameters, given the very high between-study heterogeneity observed (I^2^ = 97–99%).

Accordingly, vHIT should not be regarded as a primary diagnostic test for uncomplicated BPPV, in which high-frequency vestibular responses are usually preserved despite the presence of displaced otoconial material. Instead, its principal clinical value lies in providing complementary functional information, particularly in atypical or complex presentations, in which reduced VOR gain may indicate disturbed semicircular canal biomechanics, such as canalith jam, or associated vestibular pathology.

Future prospective multicentre studies employing standardised vHIT acquisition protocols and longitudinal assessments before and after successful canalith repositioning procedures will be essential to clarify the temporal behaviour and reversibility of VOR abnormalities in uncommon BPPV variants and to further elucidate the contribution of altered canal biomechanics to the pathophysiology of positional vertigo.

## Figures and Tables

**Figure 1:**
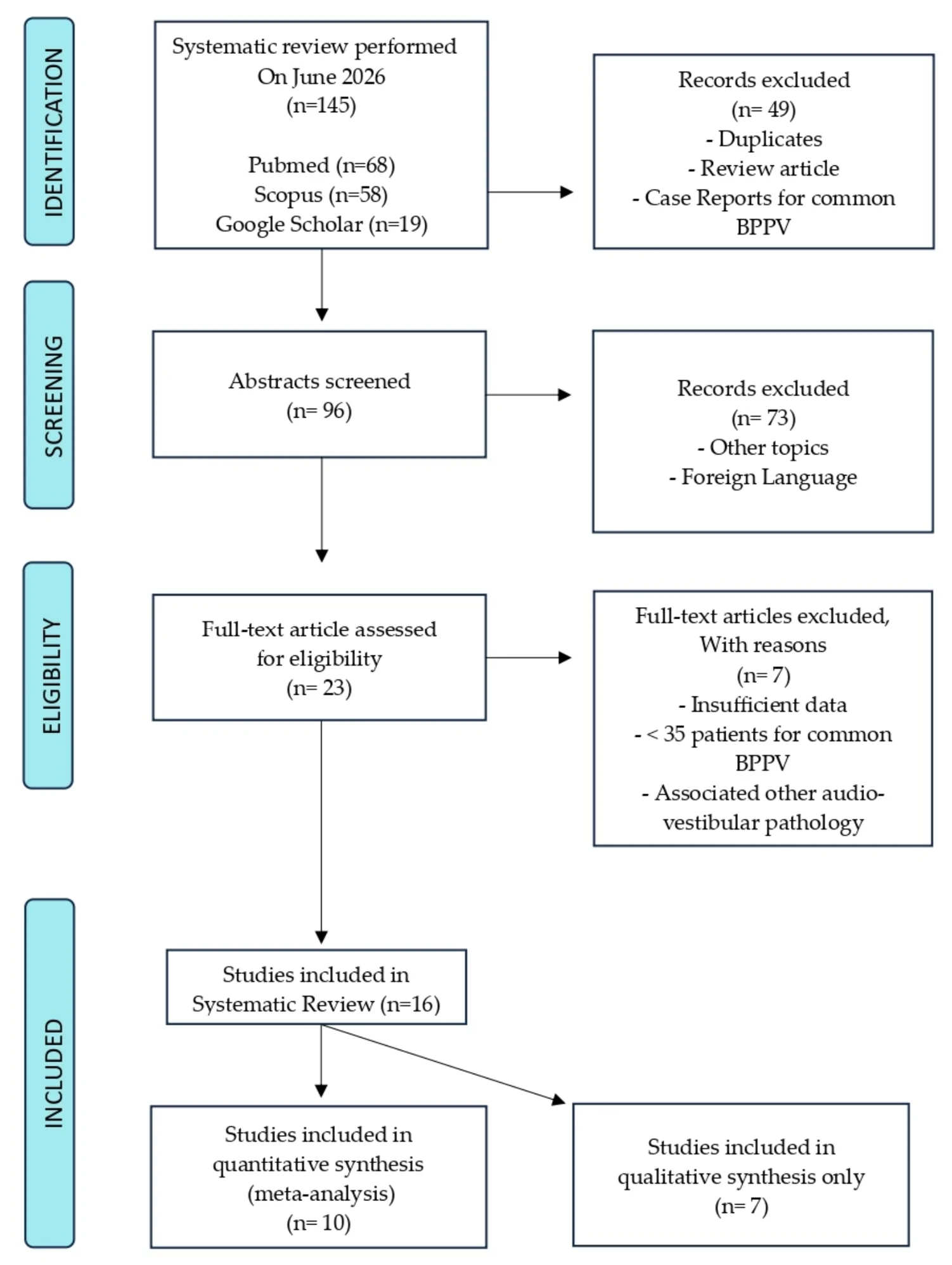
Literature evaluation and selection according to PRISMA criteria (https://www.prisma-statement.org/, URL accessed on 10 August 2026) [41]. One study was included in both the quantitative (meta-analysis) and qualitative syntheses; therefore, the sum of studies contributing to the two analyses is 17, although 16 unique studies were included overall.

**Figure 2:**
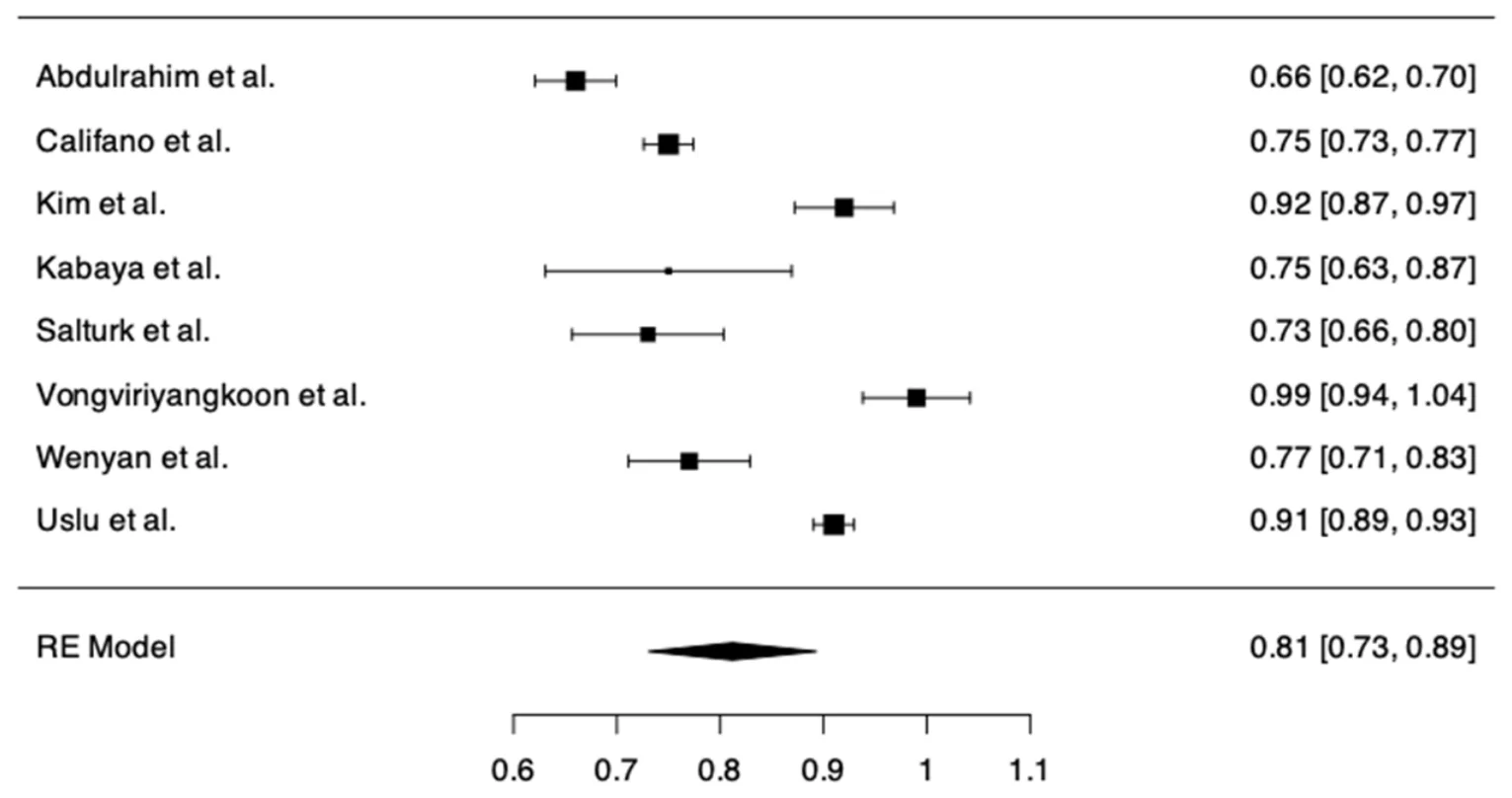
Forest plot of the meta-analysis of VOR gain measured by vHIT in patients with PSC-BPPV. The values represent VOR gain with the corresponding 95% confidence intervals [23,24,25,27,28,29,30,31]. RE Model: Random-Effects Model.

**Figure 3:**
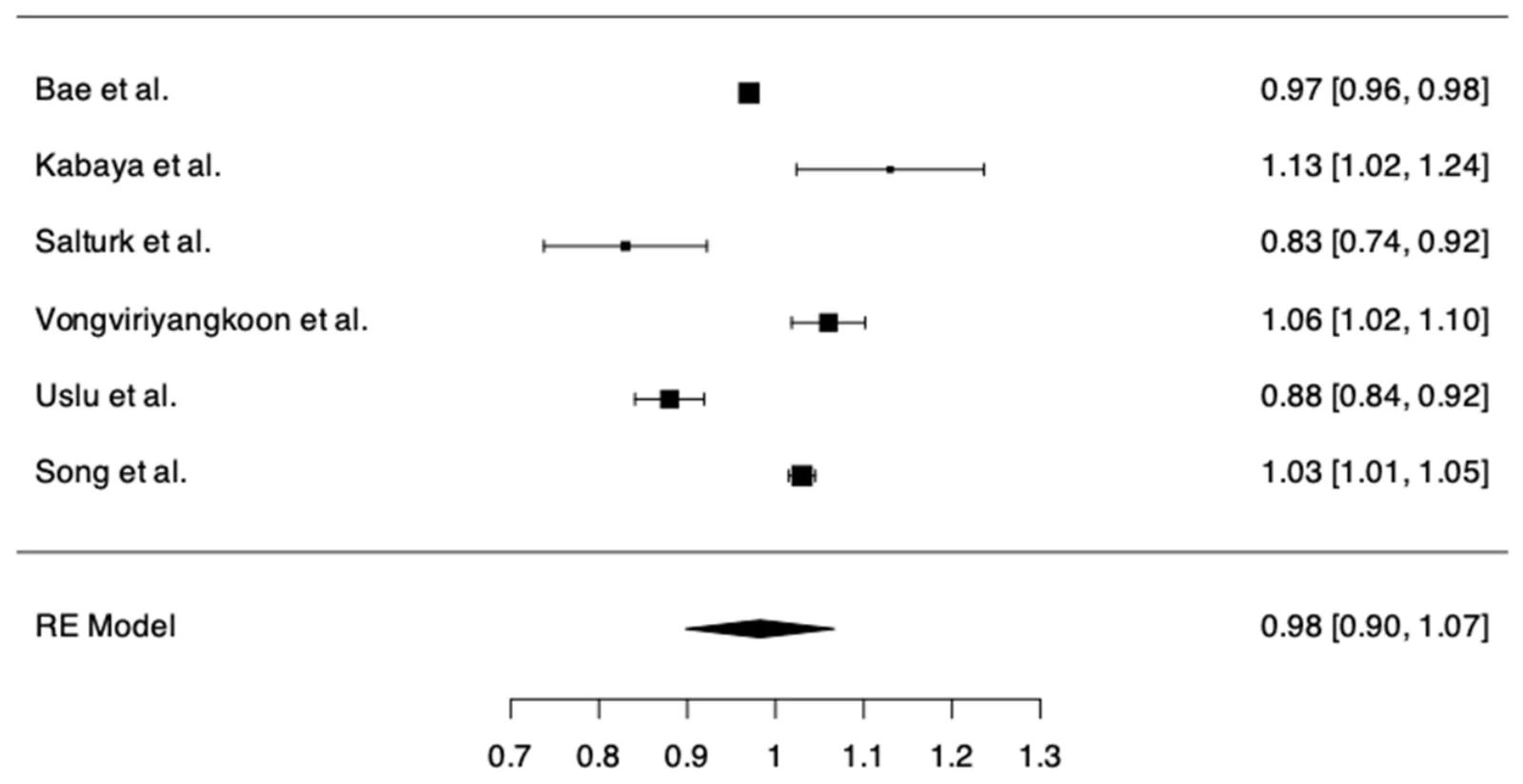
Forest plot of the meta-analysis of VOR gain measured by vHIT in patients with LSC-BPPV. The values represent VOR gain with the corresponding 95% confidence intervals [26,27,28,29,31,32]. RE Model: Random-Effects Model.

**Table 1:** Demographic, clinical, and methodological characteristics of the included studies about common BPPV.

Authors	Ref.	Year	Country	#	Sex	Age	Study
Abdulrahim et al.	[23]	2022	India	60	M: 41, F: 19	19–75 y.	O
Califano et al.	[24]	2021	Italy	150	M: 59, F: 91	19–91 y.	R
Kim et al.	[25]	2023	South Korea	50	M: 16, F: 34	25–88 y.	R
Bae et al.	[26]	2025	South Korea	101	M: 26, F: 75	49–75 y.	P
Kabaya et al.	[27]	2023	Japan	39	M: 11, F: 28	31–90 y.	R
Salturk et al.	[28]	2020	Turkey	60	M: 32, F: 28	19–59 y.	R
Vongviriyangkoon et al.	[29]	2026	Thailand	63	M: 52, F: 11	24–85 y.	P
Wenyan et al.	[30]	2024	China	167	M: 46, F: 121	18–80 y.	R
Uslu et al.	[31]	2024	Turkey	73	M: 29, F: 44	16–75 y.	P
Song et al.	[32]	2024	China	42	M:8; F:34	18-80 y.	R
Summary	-	2020–2026	-	805	M: 320, F: 485	16–91 y.	-

(O: Observational study; R: Retrospective study; P: Prospective study. #: number of patients).

**Table 2:** Summary of data extracted from the included studies regarding the diagnosis of typical BPPV and vHIT findings in the study populations.

Authors	#	Type of BPPV	Mean PSC VOR Gain at vHIT	Mean LSC VOR Gain at vHIT	Mean ASC VOR Gain at vHIT	Saccades	Timing of vHIT
Abdulrahim et al.	60	58 PSC BPPV (5/58 bilateral), 1 geotropic LSC BPPV, 1 ASC BPPV	0.66 ± 0.16	0.72	0.76	In 17/60 patients	Early unspecified phase
Califano et al.	150	150 PSC BPPV	0.75 ± 0.15 (in 10 patients < 0.55)	-	-	In 6/10 patients with gain < 0.55	Acute phase
Kim et al.	50	50 PSC BPPV	0.92 ± 0.17	-	-	-	Acute phase
Bae et al.	101	61 geotropic LSC BPPV, 40 apogeotropic LSC BPPV	-	0.97 ± 0.04 (GEO: 0.98 ± 0.04; APO: 0.96 ± 0.04)	-	-	Early unspecified phase
Kabaya et al.	39	21 PSC BPPV, 18 geotropic LSC BPPV	0.75 ± 0.28	1.13 ± 0.23	-	In 31/39 patients	Not reported
Salturk et al.	60	42 PSC BPPV (2/42 bilateral), 13 geotropic LSC BPPV, 5 apogeotropic LSC BPPV	0.73 ± 0.24	0.83 ± 0.20 (GEO: 0.85 ± 0.22; APO: 0.78 ± 0.16)	-	In 9/60 patients	Early unspecified phase
Vongviriyangkoon et al.	63	51 PSC BPPV, 11 geotropic LSC BPPV, 1 ASC BPPV	0.99 ± 0.19	1.06 ± 0.07	Not calculated	In 0/63 patients	Early unspecified phase
Wenyan et al.	167	167 PSC BPPV (122/167 canalolithiasis and 45/167 cupulolithiasis)	0.77 ± 0.38 (CANALO: 0.77 ± 0.38; CUPULO: 0.76 ± 0.39)	-	-	In 19/167 patients (14/122 with canalolithiasis and 5/45 with cupulolithiasis)	Early unspecified phase
Uslu et al.	73	57 PSC BPPV, 16 geotropic LSC BPPV	0.91 ± 0.08	0.88 ± 0.08	-	In 3/73 patients (1/57 with PSC BPPV and 2/16 with LSC BPPV)	Early unspecified phase
Song et al.	42	42 LSC cupulolithiasis	-	1.03 ± 0.05	-	-	Early unspecified phase

(#: number of patients).

**Table 3:** Demographic, clinical, and methodological characteristics of the included studies about less common BPPV.

Authors	Ref.	Year	Country	#	Sex	Age	Study
Castellucci et al.	[35]	2022	Italy	1	F: 1	56 y.	CR
Castellucci et al.	[36]	2019	Italy	1	F: 1	74 y.	CR
Perez-Fernandez et al.	[37]	2014	Spain	12	M: 4, F: 8	30–78 y.	R
Song et al.	[32]	2024	China	51	M: 9, F: 42	18–80 y.	R
Castellucci et al.	[14]	2020	Italy	3	F: 3	43–58 y.	CR
Castellucci et al.	[38]	2020	Italy	54	M: 20, F: 34	27–84 y.	R
Summary	-	2014–2024	-	122	M: 33, F: 89	18–84 y.	-

(R: Retrospective study; CR: Case report. #: number of patients).

**Table 4:** Data extracted from the included studies regarding the diagnosis of less common BPPV variants and vHIT findings. For the study by Castellucci et al. [38], only the 53 cases in which a definitive diagnosis of BPPV was established were considered.

Authors	#	Type of BPPV	Spontaneous Ny	Mean PSC VOR Gain at vHIT	Mean LSC VOR Gain at vHIT	Mean ASC VOR Gain at vHIT	Saccades
Castellucci et al.	1	ASC-BPPV	Upbeating and torsional	-	-	0.46	-
Castellucci et al.	1	LSC-canalith Jam	Horizontal left-beating	-	0.59	-	Yes
Perez-Fernandez et al.	12	12 ASC-BPPV	No	-	-	0.85 ± 0.11	-
Song et al.	51	51 LSC Light cupula	In 27/51 patients	-	1.02 ± 0.05	-	Yes
Castellucci et al.	3	2 Apogeotropic PSC-BPPV and 1 ASC-BPPV	No	0.57 ± 0.1	-	0.57	In 2 patients with Apogeotropic PSC-BPPV
Castellucci et al.	53	46 Apogeotropic PSC-BPPV and 7 ASC-BPPV	In 6/46 patients with Apogeotropic PSC-BPPV	0.62 ± 0.11	-	0.62 ± 0.09	No

(#: number of patients).

## Data Availability

The data collected in this study are available from the corresponding author upon reasonable request.

## Supplementary Materials

The following supporting information can be downloaded at: https://www.mdpi.com/article/10.3390/audiolres16050154/s1, Supplementary File: PRISMA 2020 Checklist.
